# A meta-analysis on the therapeutic efficacy of repetitive transcranial magnetic stimulation for cognitive functions in attention-deficit/hyperactivity disorders

**DOI:** 10.1186/s12888-023-05261-2

**Published:** 2023-10-17

**Authors:** Ying-Hsin Chen, Shun-Chin Liang, Cheuk-Kwan Sun, Yu-Shian Cheng, Ruu‐Fen Tzang, Hsien‐Jane Chiu, Ming-Yu Wang, Ying-Chih Cheng, Kuo-Chuan Hung

**Affiliations:** 1 Department of Emergency Medicine, Show Chwan Memorial Hospital, Changhua, Taiwan; 2 Department of Management Center, Jianan Psychiatric Center, Ministry Of Health and Welfare, Tainan, Taiwan; 3 Department of Center for General Education, University of Kun Shan, Tainan, Taiwan; 4 Department of Optometry, University of Chung Hwa of Medical Technology, Tainan, Taiwan; 5 Department of Emergency Medicine, E-Da Dachang Hospital, I-Shou University, Kaohsiung City, Taiwan; 6 School of Medicine for International Students, College of Medicine, I-Shou University, Kaohsiung City, Taiwan; 7 Department of Psychiatry, Tsyr-Huey Mental Hospital, Kaohsiung Jen-Ai’s Home, Kaohsiung City, Taiwan; 8 Department of Psychiatry, Mackay Memorial Hospital, Taipei City, Taiwan; 9 Taoyuan Psychiatric Center, Ministry of Health and Welfare, Taoyuan City, Taiwan; 10 Institute of Hospital and Health Care Administration, National Yang-Ming University, Taipei City, Taiwan; 11 Department of Psychiatry, China Medical University Hsinchu Hospital, China Medical University, Hsinchu, Taiwan; 12 Department of Health Services Administration, China Medical University, Taichung, Taiwan; 13 Institute of Epidemiology and Preventive Medicine, College of Public Health, National Taiwan University, Taipei, Taiwan; 14 Research Center of Big Data and Meta-Analysis, Wan Fang Hospital, Taipei Medical University, Taipei, Taiwan; 15 Department of Anesthesiology, Chi Mei Medical Center, Tainan City, Taiwan

**Keywords:** Repetitive transcranial magnetic stimulation, Attention-deficit/hyperactivity disorder, And cognitive functions

## Abstract

**Background:**

Therapeutic efficacies of repetitive transcranial magnetic stimulation (rTMS) for improving cognitive functions in patients with deficit/hyperactivity disorder (ADHD) remained unclear. The aim of this meta-analysis was to investigate the therapeutic efficacy of rTMS focusing on different cognitive performances.

**Methods:**

Major databases were searched electronically from inception to February 2023 by using keywords mainly “rTMS” and “ADHD” to identify randomized controlled trials (RCTs) that investigated the therapeutic efficacy of rTMS for improving cognitive functions assessed by standardized tasks in patients with ADHD. The overall effect size (ES) was calculated as standardized mean difference (SMD) based on a random effects model.

**Results:**

Meta-analysis of five RCTs with 189 participants (mean age of 32.78 and 8.53 years in adult and child/adolescent populations, respectively) demonstrated that rTMS was more effective for improving sustained attention in patients with ADHD compared with the control groups (SMD = 0.54, *p* = 0.001).Our secondary analysis also showed that rTMS was more effective for improving processing speed than the control groups (SMD = 0.59, *p* = 0.002) but not for enhancing memory or executive function.

**Conclusions:**

Our results supported the therapeutic efficacy of rTMS for improving sustained attention and processing speed. However, the limitation of available data warrants further studies to verify these findings.

**Supplementary Information:**

The online version contains supplementary material available at 10.1186/s12888-023-05261-2.

## Background

Attention deficit/hyperactivity disorder (ADHD), which commonly presents with behavioral symptoms of inattention and/or hyperactivity/impulsivity inappropriate for a child’s developmental age [1], is one of the most common neurodevelopmental disorders with a worldwide prevalence of around 5% [2]. While combined treatment with both pharmacological and behavioral interventions is commonly recommended for children and adolescents with this condition [3], challenges still exist due to concerns about side effects and stigma with medication use [4, 5], as well as a lack of satisfactory therapeutic response to behavioral interventions in a significant portion of individuals diagnosed with ADHD [6]. Moreover, because the symptoms of inattention could persist into adulthood in about 40–60% of children diagnosed with the condition [7], treatment during adulthood poses another challenge to clinicians due to comorbid presentations of mood problems and psychosocial impairments [8]. Therefore, in addition to conventional pharmacological and psychosocial interventions, therapeutic approaches of complementary alternative medicine (CAM) are also frequently sought by patients with ADHD and their caregivers [9].

Neuromodulation, that delivers stimulation to targeted regions of the brain [10, 11], has been used as a therapeutic alternative for a variety of psychiatric and neurodevelopmental disorders [12]. Two neuromodulatory approaches, namely repetitive transcranial magnetic stimulation (rTMS) and transcranial direct current stimulation (tDCS), have been shown to offer promising outcomes in the treatment of ADHD [12]. Although a previous meta-analysis attempted to investigate the therapeutic efficacy of both rTMS and tDCS, the treatment benefits of rTMS remains inconclusive due to lack of available information [12]. Nevertheless, that study and another meta-analytical investigation both demonstrated preliminary evidence in support of the therapeutic potential of tDCS for improving some cognitive functions such as processing speed and inhibitory controls [12, 13]. However, the positive findings were only limited to subgroups of selected studies (i.e., tDCS focusing only on the left dorsolateral prefrontal cortex, or in children) [12], and none of them showed the efficacy of tDCS for improving attentional functions [12, 13]. In addition, the therapeutic efficacy of rTMS, which involves induction of a more focal stimulation than that with tDCS [14], has not been systematically reviewed for ADHD-related symptoms and cognitive deficits [12].

The potential therapeutic effects of rTMS are theoretically based on neurostimulation targeting areas of hypofunction in specific brain regions responsible for different cognitive functions [12]. While the exact mechanism underlying the therapeutic effects of rTMS remains unclear, several studies have reported an association of rTMS treatment with an induction of brain dopamine release [15] and an increase in synaptic plasticity [16, 17]. Despite the choice of the prefrontal cortex (PFC) as the target region for rTMS stimulation in previous randomized controlled trials (RCTs) [18–20], taking into consideration its critical role in attentional as well as many other important cognitive functions [21], their findings regarding the therapeutic effectiveness of rTMS varied with different core symptoms of ADHD [18–20]. For instance, one study showed an efficacy of rTMS only for the symptoms of inattention but not those of hyperactivity/impulsivity [18]. That study further demonstrated that rTMS was effective for improving inattention when targeting the right PFC but not the left PFC [18]. The finding was consistent with that of a previous study showing predominant underactivation of brain regions on the right hemisphere rather than those on the left side [22]. On the other hand, the previous report of an association between the left brain hemisphere (i.e., left caudate head) and impulsivity suppression [22], may explain the efficacy of tDCS targeting the left dorsolateral PFC for better inhibitory control in previous meta-analyses [12, 13]. These findings, therefore, highlighted the probability that the therapeutic effects of rTMS may vary when targeting different brain regions [18].

Informant bias remains an unabated issue when assessing the therapeutic outcomes for children with ADHD [23]. Indeed, subjective rating of clinical symptoms not only may be more susceptible to informant biases [24], but also could not provide detailed information regarding specific neuro-cognitive functions [25]. Therefore, objective evaluation of different cognitive functions with cognitive tasks may be more reliable in the assessment of the therapeutic efficacy of rTMS in patients with ADHD.

The aims of present meta-analysis were, therefore, to investigate the therapeutic efficacy of rTMS focusing on different cognitive performances measured by standardized tasks as well as to identify possible factors (e.g., different locations of stimulation) that may influence its therapeutic outcomes in patients with ADHD.

## Methods

### Protocol and registration

The current meta-analysis was conducted according to the Preferred Reporting Items for Systematic Reviews and Meta-Analyses (PRISMA) guidelines [26]. The protocol of this study was registered with the international prospective register of systematic reviews (PROSPERO CRD42023398406).

### Search strategy and selection criteria

We searched major databases including the PubMed, Embase, Cochrane CENTRAL, and ClinicalTrials.gov from inception to February 2023 without any limitation on language for randomized controlled trials (RCTs) that investigated the therapeutic efficacy of rTMS for improving cognitive functions in patients diagnosed with ADHD. The main keywords used were “rTMS” and “ADHD”. Our search strategies with keywords used for each database are provided in eTable 1. To expand our search, we also attempted to identify any relevant studies from the reference lists of important reviews on this topic. The PICO criteria (i.e., population, intervention, comparator, and outcomes) for study inclusion were (1) Population: RCTs of patients diagnosed with ADHD, (2) Intervention: rTMS used either as monotherapy or in combination with others, (3) Comparator: waitlist, treatment as usual, sham stimulation or interventions other than rTMS, and (4) Outcome: standardized tests for assessment of attentional function and other important cognitive functions such as memory. Studies that did not focus on patients diagnosed with ADHD or used interventions not related to rTMS were excluded.

### Data extraction and quality assessment

Two independent authors (Sun CK and Cheng CM) screened the titles/abstracts by using pre-determined search strategies (eTable 1). Full texts of relevant studies were then reviewed to identify eligible studies for study inclusion. The kappa coefficient was used to examine inter-rater reliability [27]. Any disagreement about eligibility of studies was resolved by discussion with a third author (Liang SC). Studies characteristics (e.g., number of participants, gender proportion, number of rTMS sessions, and follow-up duration) and publication-specific details (e.g. authors and date of publication) were then extracted. Electronic emails were sent to corresponding authors in case of missing data. Cochrane's “risk of bias” assessment tool [28] was used to rate the quality of the included studies with the risk of bias being classified as “low”, “unclear”, or “high” in following seven categories, namely random sequence generation, allocation concealment, performance bias, detection bias, attrition bias, reporting bias, and one additional category for identifying other important biases. Finally, the certainty of evidence of the results from the present meta-analysis was rated according to the Grading of Recommendations Assessment, Development and Evaluation (GRADE) framework [29].

### Primary and secondary outcomes

The primary outcome was improvement in sustained attention measured by standardized tests such as continuous performance test (CPT). The secondary outcomes included improvements in other important cognitive domains (e.g., working memory and processing speeds) measured by standardized neuropsychological tests (e.g., digit span).

### Data synthesis and analysis

The overall effect size (ES) was calculated as standardized mean difference (SMD) with 95% confidence interval (CI) for continuous data and odds ratios (ORs) with 95% CI for categorical data. The cutoff for statistical significance was defined by a *p* value less than 0.05. To provide a more generalizable result by including the same outcome of interest assessed by different assessment tools, a random effects model was used. For data analysis, we chose Review Manager 5 (RevMan 5.4; Copenhagen: The Nordic Cochrane Center, The Cochrane Collaboration, 2014), in which the Mantel–Haenszel (MH) method was selected for odds ratio and generic inverse-variance method for outcomes of continuous variables. If a study provided more than one dataset with the rTMS treatment group (i.e. rTMS targeting different brain locations), the results from different treatment arms were combined to give a single SMD. We also conducted subgroup analysis on rTMS targeting different brain locations. Reliability of each outcome of interest was tested through a leave-one-out sensitivity analysis. I-squared statistics (low: ≤ 50%; moderate: 50% to 75%, high: ≥ 75%) was used to assess the heterogeneity of eligible studies. Finally, visual inspection of a funnel plot was used to assess the risk of publication bias.

## Results

### Study selection and characteristics of included studies

**Fig. 1:**
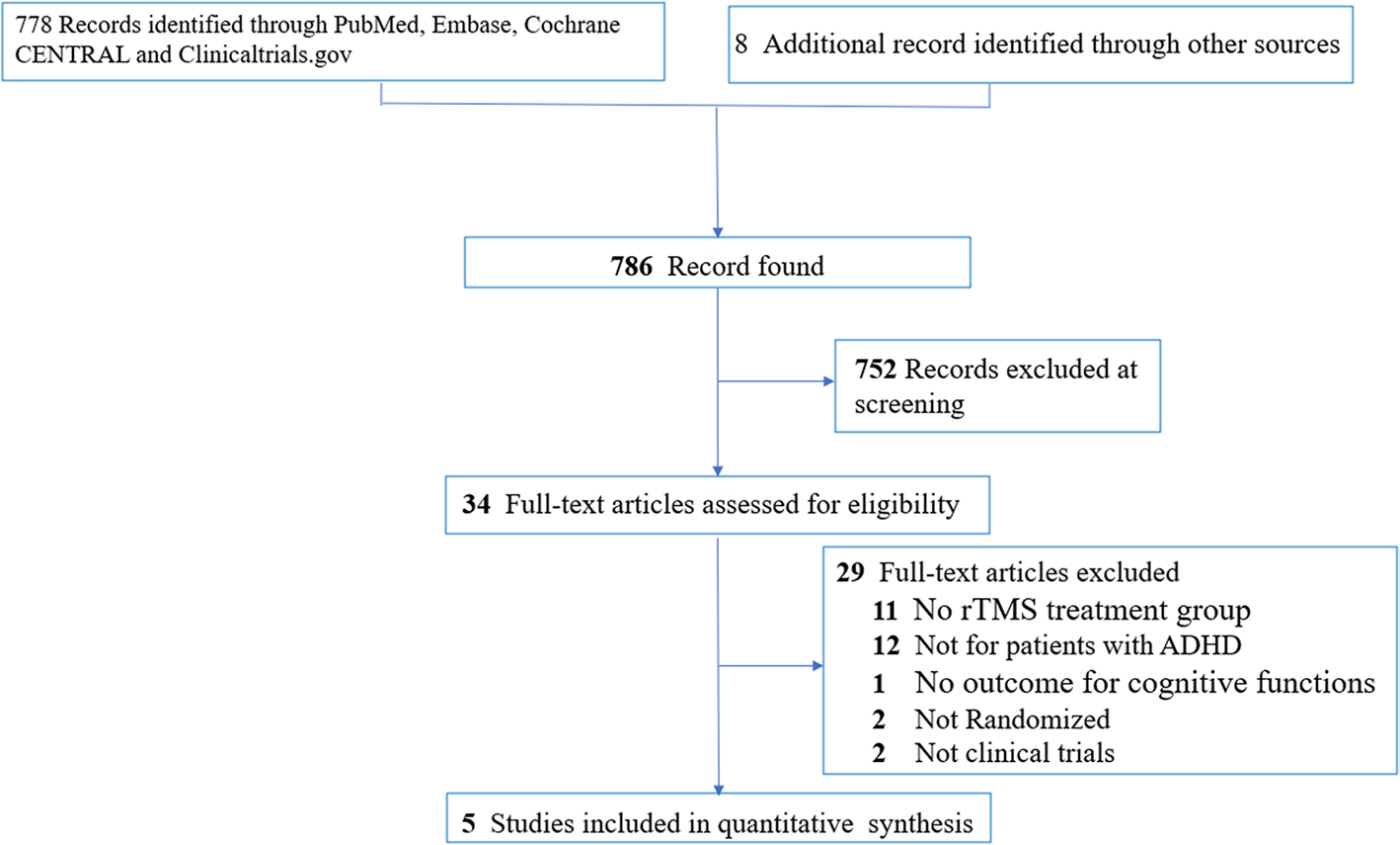
PRISMA diagram of identifying eligible studies. ADHD: Attention Deficit/Hyperactivity Disorder; rTMS: repetitive transcranial magnetic stimulation

Figure 1 shows the flowchart of the study selection process according to the PRISMA statement [26]. Of the 786 articles identified from the PubMed, Embase, Cochrane CENTRAL, and ClinicalTrials.gov through electronic search, as well as from other sources of important review articles, 34 were selected for full text review after exclusion of 752 articles through screening of title and abstract. 29 articles were excluded for different reasons (eTable 2) and finally five RCTs with 189 participants were included in this meta-analysis [18–20, 30, 31]. With regard to the eligibility of the included studies in the present meta-analysis, there was no disagreement between the two reviewers (kappa coefficient = 1).

Table 1 summarizes the characteristics of the eligible studies. While four of the five included studies only included adults [18–20, 30, 31], the other study included only children and adolescents [30]. In terms of control group, four of the five studies used sham rTMS as control groups [18–20] and the other compared rTMS in combination with atomoxetine (ATX) versus ATX treatment only [30]. The mean ages of participants in the four studies that only included adults [18–20, 31] and the other study that recruited only children and adolescents [30] were 32.78 (range = 18–65 years) and 8.53 (range = 6–13 years), respectively. The proportion of females ranged between 15.63% and 82.76%. The participants received a median of 20 rTMS treatment sessions (range = 15–30 sessions) with a median duration of follow-up being four weeks (range = 3–6 weeks). Regarding the use of medications for ADHD, three studies prohibited the use of any medications for ADHD [18–20], one did not provide such information [31] and one used ATX for all participants [30]. Of the five included studies, three were conducted in Israel [18–20], one in China [30] and one in the USA [31].

**Table 1:** Summary of characteristics of studies in the current meta-analysis

Study (year)	Diagnosis (Criteria)	Design	Comparison	N	Duration (weeks)	Frequency (Hz)	Intensity of stimulation	Number of sessions	Outcome		Medications	Mean age (years)	Female (%)	Country
Bleich-Cohen M (2021) [18]	ADHD (DSM-5)	RCT	rTMS: rPFC	24	3	18	120% of RMT	15	Sustain attention: Mindstreams attention Working memory: Mindstreams memory Processing speed: Mindstreams information Processing Executive control: Mindstreams Executive function		Allowed only SSRI and SNRI Not allowed neuroleptics or psychostimulant	35.1 (18–60)	34.7	Israel
			rTMS: lPFC	22										
			Sham	16										
Alyagon U (2020) [20]	ADHD(DSM-5)	RCT	rTMS: rPFC	15	3	18	120% of RMT	15	Sustain attention: Mindstreams attention Working memory: Mindstreams memory Processing speed: Mindstreams information Processing Executive control: Mindstreams Executive function	Not allowed psychostimulant		27.1 (21–64)	82.76	Israel
			Sham	14										
Cao P (2018) [30]	ADHD (DSM-5)	RCT	rTMS: rPFC + ATX	21	6	10	100% of RMT	30	Sustain attention: CPT Working memory: Digit span Processing speed: WISC – coding Executive control: IGT	All participants used atomoxetine		8.53 (6–13)	15.63	China
			ATX only	19										
Paz Y (2018) [19]	ADHD (DSM-5)	RCT	rTMS: bil.PFC	12	4	18	120% of RMT	20	Sustain attention: T.O.V.A	Not allowed any neuropsychiatric medications		31.6 (≥ 18)	46.15	Israel
			Sham	14										
Loughead J NCT03663179	ADHD (SCID-5)	RCT	rTMS: lPFC	18	4	10	120% of RMT	20	Sustain attention: CPT	N/A		34.4 (18–65)	33	USA
			Sham	14										

*Abbreviations:* *ADHD* Attention-deficit/hyperactivity disorder, *ATX* Atomoxetine, *bil.PFC* Bilateral prefrontal cortex, *CPT* Continuous performance test, *DSM-5* Diagnostic and statistical manual of mental disorders fifth edition, *IGT* Iowa Gambling Task, *lPFC* left prefrontal cortex, *N* Number, *RCT* Randomized controlled trial, *RMT* Rest motor threshold, *rPFC* Right prefrontal cortex, rtms repetitive transcranial magnetic stimulation, *scid-5* the Structured Clinical Interview for *DSM-5* SNRI serotonin and norepinephrine reuptake inhibitors, *SSRI* Serotonin reuptake inhibitor, *T.O.V.A* Test of Variables of Attention, *WISC* Wechsler Intelligence Scale for Children

### Risk of bias assessment

**Fig. 2:**
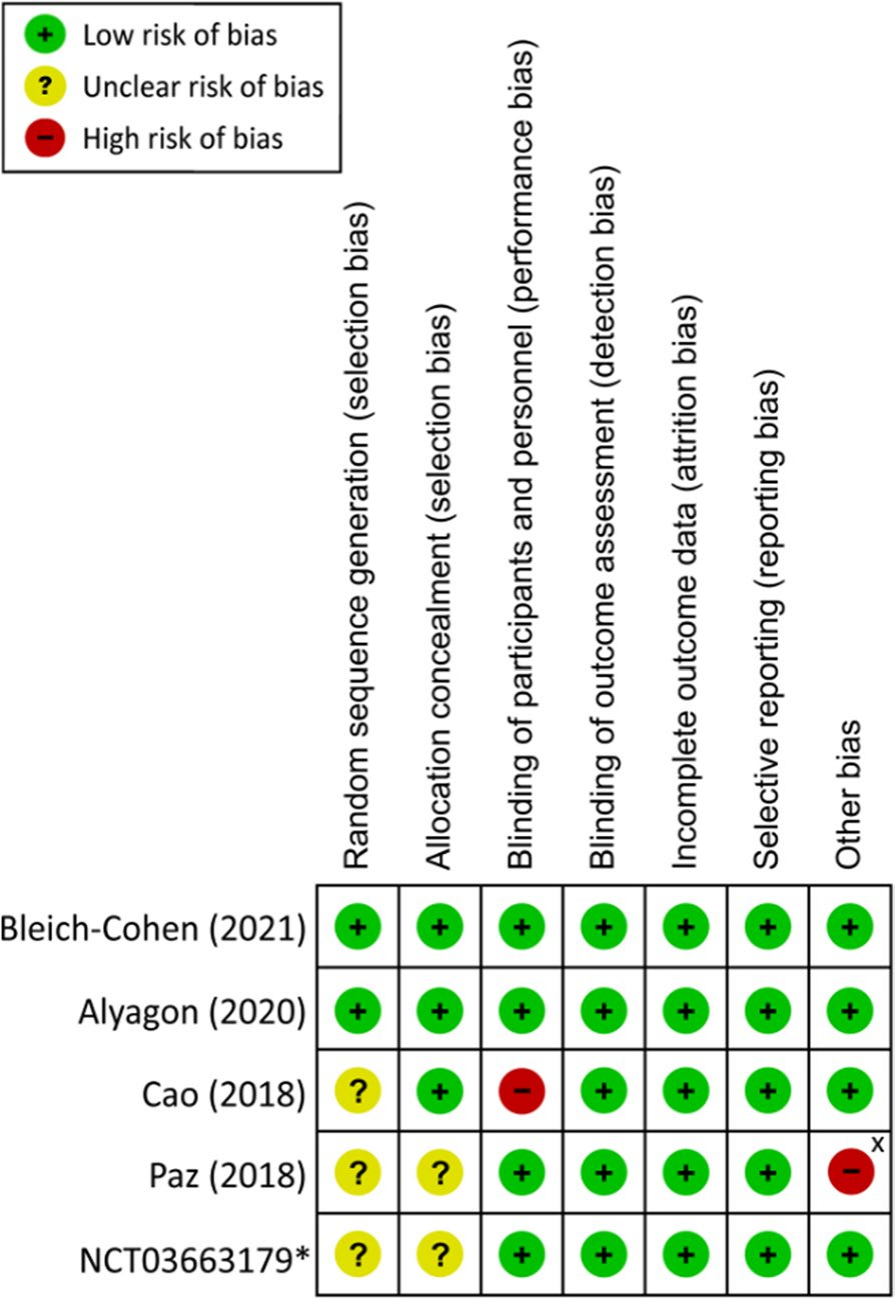
Risk of bias for eligible studies ^*^Study from clinicaltrials.gov ^X^ Study received no financial support from private companies

According to the Cochrane Collaboration's tool for risk of bias assessing, the risks of performance and detection biases were deemed low in four studies as sham rTMS was used in their control groups, while one study that did not use sham rTMS was considered at high risk of performance bias [30]. Moreover, a high risk of bias in the “other bias” category was given to one study that received funding from a private company [19]. In addition, the risks of biases for random sequencing and allocation concealment were deemed unclear in three and two studies, respectively, due to lack of relevant information. The overall risks of biases in most categories were considered low among the eligible studies (Fig. 2).

## Results of syntheses

### Primary outcome

**Fig. 3:**
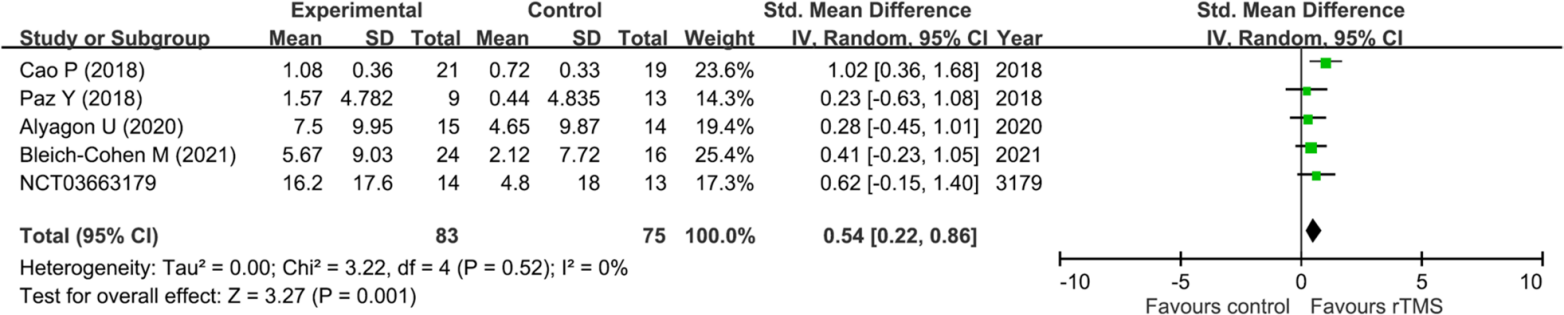
Forest plot of effect size for comparing the difference in the improvement of sustained attention between rTMS and control groups CI: confidence interval; rTMS: repetitive transcranial magnetic stimulation; Std: standardized; SE: standard error

Our results showed that rTMS was more effective for improving sustained attention in patients with ADHD compared with the control groups (SMD = 0.54, 95% CI: 0.22 to 0.86, *p* = 0.001, five studies with 149 participants) (Fig. 3). The results of leave-one-out sensitivity analysis remained unchanged, suggesting the robustness of this finding. There was also no significant heterogeneity (I^2^ = 0%, Tau^2^ = 0.00 and *p* = 0.52) and no notable asymmetry on visual inspection of a funnel plot (eFigure 1).

**Fig. 4:**
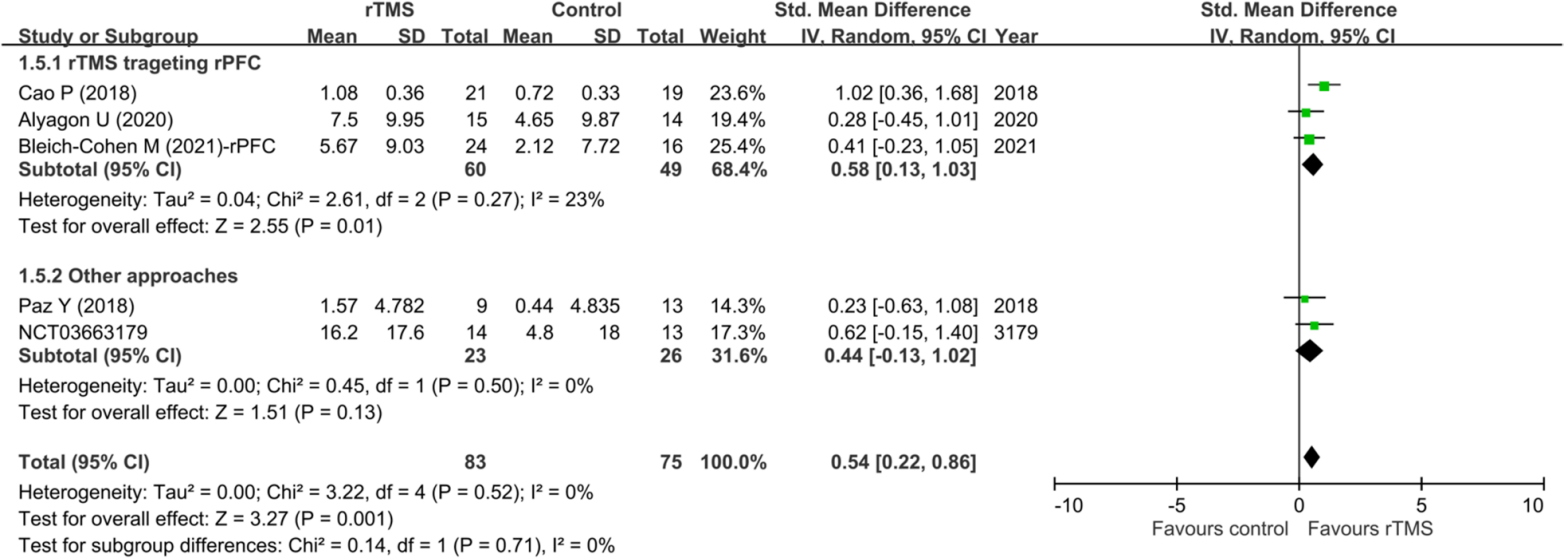
Subgroup analysis—forest plot of effect sizes in subgroup of studies using rTMS focusing on rPFC versus those using other approaches CI: confidence interval; rPFC: right prefrontal cortex; rTMS: repetitive transcranial magnetic stimulation; Std: standardized; SE: standard error

A subgroup analysis focusing on the efficacy of rTMS targeting different locations of the brain revealed a significant therapeutic benefit of targeting rPFC (SMD = 0.58, 95% CI: 0.13 to 1.03, *p* = 0.01, three studies with 109 participants), but a lack of efficacy by targeting other locations (i.e., bilateral PFC or only lPFC) (SMD = 0.44, 95% CI: -0.13 to 1.02, *p* = 0.13, two studies with 49 participants) compared with their respective controls. Nevertheless, there was no significant difference in therapeutic efficacy of rTMS between studies that targeted rPFC and those targeting other locations (*p* = 0.71) (Fig. 4).

### Secondary outcomes

Our secondary outcome analysis showed that rTMS was more effective only for improving processing speed than the control groups (SMD = 0.59, 95% CI: 0.22 to 0.96, *p* = 0.002, three studies with 131 participants) but not for enhancing memory (SMD = 0.18% CI: -0.52 to 0.87, *p* = 0.62, three studies with 131 participants) or executive function (SMD = 0.23, 95% CI: -0.24 to 0.71, *p* = 0.34, three studies with 131 participants) (eFigure 2 to 4). There was no significant heterogeneity (I^2^ = 0%, Tau^2^ = 0.00 and *p* = 0.99) and leave-one-out sensitivity analysis remained unchanged in the result for processing speed, suggesting stability of this finding. On the other hand, the leave-one-out sensitivity analysis demonstrated significantly better therapeutic effects of rTMS for improving memory and executive function after excluding the study by Bleich-Cohen M et al. [18]. Significant heterogeneity was found in the result of memory (I^2^ = 71%, Tau^2^ = 0.27, *p* = 0.03) but not in that of executive function (I^2^ = 40%, Tau^2^ = 0.07, *p* = 0.19). There was no obvious asymmetry on visual inspection of funnel plots in our secondary analyses (eFigure 5–7).

### Certainty of evidence

Overall, the certainty of evidence for all outcomes according to the GRADE assessment was downgraded mainly for serious imprecision and publication biases due to the limited number of eligible trials. While the certainty of evidence for sustained attention and processing speed was down-graded to low, the certainty was further down-graded to very low for memory and executive function because of additional problems with inconsistency. Detailed information about the certainty of evidence of individual outcomes is provided in eTable 3.

## Discussion

Although treatments using brain stimulation have been reported to improve cognitive functions and attention associated with several psychiatric and neurodevelopmental disorders [32], evidence supporting the therapeutic effect of rTMS on attentional performance was still limited [12]. To our best knowledge, our meta-analysis is the first to investigate the therapeutic efficacy of rTMS for improving cognitive functions in patients with ADHD. Our results demonstrated more significant improvements in sustained attention and processing speed but not in memory and executive function among ADHD patients treated with rTMS compared with those in the control groups. On the other hand, we found no significant difference between rTMS focusing particularly on rPFC and that targeting other locations (e.g., both right and left PFC or lPFC only). Nevertheless, the soundness of evidence derived from the current study warrants elucidation from further clinical investigations given the limited number of eligible studies (*n* = 5, total sample size = 189).

The result of our primary outcome showed that treatment with rTMS was associated with a more significant improvement in sustained attention than in controls when assessed by mindstreams battery and CPT, which were computerized versions of standardized attentional tests. Given that standardized attentional tests may provide a more objective assessment, our results were less susceptible to informant bias [24], which has been reported to be an significant issue in studies using questionnaire-based evaluation for patients with ADHD [33]. Moreover, assessment of cognitive function by using standardized attentional tests may better reflect improvement in neurocognitive functioning (i.e., endophenotype) [25] that should theoretically precede behavioral improvements (i.e., phenotype) reflected by questionnaire-based rating scales. Therefore, based on standardized attentional tests, our results may be more sensitive to the detection of the therapeutic effects of rTMS on neurocognitive functions compared to questionnaire-based studies. In addition to a minimization of informant bias, low risk of performance bias in our results was also evident as reflected by the consistency of our primary outcome (i.e., improvement in sustained attention) on leave-one-out sensitivity analysis after excluding the only study that did not use sham rTMS [30]. Overall, our results supported the therapeutic efficacy of rTMS for improving attentional function in patients with ADHD.

Despite unclear mechanisms underlying the therapeutic effects of rTMS on neurocognitive functions, previous studies have demonstrated an association of rTMS treatment with induction of dopamine release in the brain [15] and enhancement of synaptic neuroplasticity [16, 17]. It is also well-reported that different regions of the brain are linked to various cognitive functions [34]; therefore, rTMS targeting specific brain regions to enhance a particular neurocognitive function seems a reasonable therapeutic approach [32]. Indeed, previous evidence suggested a stronger correlation of cognitive dysfunction with the right hemisphere compared to that on the left in patients with ADHD [35, 36], highlighting the importance of investigating the possible influence of targeting different brain regions on the therapeutic efficacy of rTMS. Nevertheless, notwithstanding the report of a stronger association between attentional function and the right dorsal prefrontal cortex (rDLPFC) in a prior meta-analysis of brain imaging data from patients with ADHD, the same study also suggested that interference inhibition was more related to the left caudate head [36]. Taking into account the need for inhibiting irrelevant stimuli to maintain concentration [37], both left and right hemispheres of the brain may have important albeit different roles to play in sustaining attention. Consistently, targeting the left side of the brain was found to enhance inhibitory control and processing speed in patients with ADHD in previous meta-analyses investigating tDCS, which is another electrical stimulation approach [12, 13]. However, we could only study the therapeutic efficacy of rTMS targeting rPFC but not those focusing on the left hemisphere due to the limited number of RCTs targeting the left hemisphere (*n* = 1) [31]. While our subgroup analysis showed similar therapeutic efficacies for improving sustained attention between rTMS targeting rPFC and other approaches (e.g., those targeting both right and left PFC), we were unable to study inhibitory controls due to a lack of available outcome data from the eligible studies. Therefore, although our subgroup analysis failed to demonstrate significant differences in therapeutic efficacy of rTMS for improving sustained attention through targeting different brain regions, we could not rule out the possibility that targeting different brain regions may enhance different aspects of attentional functions.

With regard to our secondary outcomes, the results were only representative of the therapeutic efficacy of rTMS targeting rPFC because only studies focusing on rPFC provided analyzable data. Our findings showed that the therapeutic efficacy of rTMS was only significantly better than that of the controls in terms of processing speed, but not in memory and executive functions. However, leave-one-out sensitivity analysis showed significantly superior therapeutic efficacies of rTMS for improving memory and executive function after excluding the study by Bleich-Cohen M et al. [18]. Nevertheless, the result was derived from only two remaining studies [20, 30]. Besides, a significant heterogeneity was noted in the result for memory (I^2^ = 71%, *p* = 0.03). Therefore, the results of our secondary outcomes regarding memory and executive function were not robust and warranted further studies for elucidation. On the other hand, our finding of rTMS-related improvement in processing speed was consistent with that of another meta-analysis on ADHD patients treated with tDCS [13]. Nevertheless, the positive result on processing speed improvement in that meta-analysis came from tDCS targeting the left PFC instead of the right as reflected in our finding. In addition, several fMRI studies also found that processing speed may be more associated with the left PFC [38, 39]. On the other hand, some brain-imaging studies have shown that both right and left PFC may play a role in processing speed [40, 41]; therefore targeting rPFC may still be a therapeutic option for improving processing speed. The limited available data precluded our investigation into the difference in therapeutic efficacy between rTMS targeting the right PFC and that targeting the left PFC. Besides, because rTMS-elicited changes in processing speed may be attributed to improvements in different domains of cognitive functions, further studies are warranted to shed light on these issues.

Despite the design of the current study to minimize informant bias and provide more reliable measures of neurocognitive functions, there were several limitations. First, notwithstanding the moderate ES regarding the improvement in sustained attention related to rTMS treatment without significant heterogeneity or a change in result on sensitivity analysis, the significance of our finding may still be obscured by the limited number of included studies and participants. In addition, the measurement of heterogeneity by using I-squared statistic is prone to bias, especially when the numbers of studies are small. Second, while the majority of our included studies recruited adult participants diagnosed with ADHD, only one targeted children and adolescents [30]. Therefore, taking into account the difference in stages of brain development between adults and children/adolescents [42], the results of this meta-analysis may not be generalizable to the younger populations. For instance, one study still found differences in the areas of brain activation between the two populations during tasks for verbal working memory despite their overall similarity [42]. Third, despite previous evidence showing an association between ADHD and the right PFC [35, 36], we were unable to directly compare the treatment efficacies between rTMS targeting the right PFC and that targeting the left side. Nevertheless, our subgroup analysis demonstrated quite similar ES between rTMS only targeting rPFC and rTMS targeting other locations (i.e., both left and right PFC or left PFC). Given that inhibitory control, which is quite important for sustaining attention, has been reported to be more related to the left hemisphere [36], current evidence is still insufficient to provide more precise information about the influence of targeting different brain regions on different subcategories of cognitive functions. Fourth, the limited geographic scope of our included studies (i.e., three from Israel [18–20], one from China [30], and one from USA) may restrict extrapolation of our findings to populations of different ethnic and cultural backgrounds. Fifth, our results derived from only objective assessments of cognitive functions may not reflect patients’ perception or subjective feelings from important others (i.e. parents or teachers). Nevertheless, results from objective evaluations of cognitive functions may be better indicators of improvement in neurocognitive functions compared with those acquired with subjective tests [25]. Finally, we were unable to conduct subgroup analyses or meta-regression focusing on other important factors (e.g., age or number of treatment sessions) that could affect the therapeutic outcomes of rTMS due to the limited number of RCTs included in current meta-analysis.

## Conclusions

Our results supported the therapeutic efficacy of rTMS for certain cognitive functions including sustained attention and processing speed, but showed no difference between rTMS focusing on rPFC and that targeting other locations (i.e., both left and right PFC or the left PFC) in individuals diagnosed with ADHD. However, the limitation of available data warrants further studies to elucidate the correlation between the selection of brain regions for rTMS targeting and its therapeutic efficacy for different sub-categories of cognitive functions.

### Supplementary Information

**Additional file 1.**

## Data Availability

The datasets used and/or analyzed during the current study are available from the corresponding author on reasonable request.
