# Supplementary material for: A meta-analysis on the therapeutic efficacy of repetitive transcranial magnetic stimulation for cognitive functions in attention-deficit/hyperactivity disorders
Source: BMC Psychiatry. 2023 Oct 17;23:756. doi: 10.1186/s12888-023-05261-2 (PMC10580630; doi:10.1186/s12888-023-05261-2)

eTable 1. Applied keywords and the search results from each database

Database	Keywords	Filter	Date (yyyy/mm/dd)	Result
PubMed	(adhd OR hkd OR add OR Attention deficit hyperactivity disorder OR attention deficit OR hyperactivity OR inattentive OR impulsivity) and (rTMS OR repetitive transcranial magnetic stimulation OR brain stimulation)	RCT	2023/02/26	258
Embase	(adhd OR hkd OR add OR Attention deficit hyperactivity disorder OR attention deficit OR hyperactivity OR inattentive OR impulsivity) and (rTMS OR repetitive transcranial magnetic stimulation OR brain stimulation)	RCT	2023/02/26	173
Cochrane CENTRAL	(adhd OR hkd OR add OR Attention deficit hyperactivity disorder OR attention deficit OR hyperactivity OR inattentive OR impulsivity) and (rTMS OR repetitive transcranial magnetic stimulation)	Trials	2023/02/26	340
ClinicalTrials.gov	(Attention deficit hyperactivity disorder) and (repetitive transcranial magnetic stimulation)	NA	2023/02/26	7

Abbreviations: NA, not applied; RCT, randomized controlled trial

eTable 2 Reasons for study exclusion

Reason	Number of excluded studies	References
No rTMS treatment group	11	[1-11]
Not for patients with ADHD	12	[12-23]
No outcome for cognitive functions	2	[24, 25]
Not Randomized	2	[26, 27]
Not clinical trials	2	[28, 29]

rTMS repetitive transcranial magnetic stimulation

ADHD Attention deficit hyperactivity disorder

RCT randomized controlled trials

References

[1] Sotnikova A, Soff C, Tagliazucchi E, Becker K, Siniatchkin M. Transcranial Direct Current Stimulation Modulates Neuronal Networks in Attention Deficit Hyperactivity Disorder. Brain Topogr. 2017;30(5):656-72. https://doi.org/10.1007/s10548-017-0552-4.

[2] Gilbert DL, Wang Z, Sallee FR, Ridel KR, Merhar S, Zhang J, et al. Dopamine transporter genotype influences the physiological response to medication in ADHD. Brain. 2006;129(Pt 8):2038-46. https://doi.org/10.1093/brain/awl147.

[3] Gilbert DL, Ridel KR, Sallee FR, Zhang J, Lipps TD, Wassermann EM. Comparison of the inhibitory and excitatory effects of ADHD medications methylphenidate and atomoxetine on motor cortex. Neuropsychopharmacology. 2006;31(2):442-9. https://doi.org/10.1038/sj.npp.1300806.

[4] Barham H, Büyükgök D, Aksu S, Soyata AZ, Bulut G, Eskicioğlu G, et al. Evidence for modulation of planning and working memory capacities by transcranial direct current stimulation in a sample of adults with attention deficit hyperactivity disorder. Neurosci Lett. 2022;790:136883. https://doi.org/10.1016/j.neulet.2022.136883.

[5] Leffa DT, Grevet EH, Bau CHD, Schneider M, Ferrazza CP, da Silva RF, et al. Transcranial Direct Current Stimulation vs Sham for the Treatment of Inattention in Adults With Attention-Deficit/Hyperactivity Disorder: The TUNED Randomized Clinical Trial. JAMA Psychiatry. 2022;79(9):847-56. https://doi.org/10.1001/jamapsychiatry.2022.2055.

[6] Zhuo L, Zhao X, Zhai Y, Zhao B, Tian L, Zhang Y, et al. Transcutaneous electrical acupoint stimulation for children with attention-deficit/hyperactivity disorder: a randomized clinical trial. Transl Psychiatry. 2022;12(1):165. https://doi.org/10.1038/s41398-022-01914-0.

[7] Dubreuil-Vall L, Gomez-Bernal F, Villegas AC, Cirillo P, Surman C, Ruffini G, et al. Transcranial Direct Current Stimulation to the Left Dorsolateral Prefrontal Cortex Improves Cognitive Control in Patients With Attention-Deficit/Hyperactivity Disorder: A Randomized Behavioral and Neurophysiological Study. Biol Psychiatry Cogn Neurosci Neuroimaging. 2021;6(4):439-48. https://doi.org/10.1016/j.bpsc.2020.11.006.

[8] Bell SB, Turner B, Sawaki L, DeWall N. When brain stimulation backfires: the effects of prefrontal cortex stimulation on impulsivity. Soc Cogn Affect Neurosci. 2022;17(1):101-8. https://doi.org/10.1093/scan/nsaa049.

[9] McGough JJ, Sturm A, Cowen J, Tung K, Salgari GC, Leuchter AF, et al. Double-Blind, Sham-Controlled, Pilot Study of Trigeminal Nerve Stimulation for Attention-Deficit/Hyperactivity Disorder. J Am Acad Child Adolesc Psychiatry. 2019;58(4):403-11.e3. https://doi.org/10.1016/j.jaac.2018.11.013.

[10] Allenby C, Falcone M, Bernardo L, Wileyto EP, Rostain A, Ramsay JR, et al. Transcranial direct current brain stimulation decreases impulsivity in ADHD. Brain Stimul. 2018;11(5):974-81. https://doi.org/10.1016/j.brs.2018.04.016.

[11] Gilmore CS, Dickmann PJ, Nelson BG, Lamberty GJ, Lim KO. Transcranial Direct Current Stimulation (tDCS) paired with a decision-making task reduces risk-taking in a clinically impulsive sample. Brain Stimul. 2018;11(2):302-9. https://doi.org/10.1016/j.brs.2017.11.011.

[12] Wu SW, Maloney T, Gilbert DL, Dixon SG, Horn PS, Huddleston DA, et al. Functional MRI-navigated repetitive transcranial magnetic stimulation over supplementary motor area in chronic tic disorders. Brain Stimul. 2014;7(2):212-8. https://doi.org/10.1016/j.brs.2013.10.005.

[13] Ni HC, Chen YL, Chao YP, Wu CT, Wu YY, Liang SH, et al. Intermittent theta burst stimulation over the posterior superior temporal sulcus for children with autism spectrum disorder: A 4-week randomized blinded controlled trial followed by another 4-week open-label intervention. Autism. 2021;25(5):1279-94. https://doi.org/10.1177/1362361321990534.

[14] Kang J, Li X, Casanova MF, Sokhadze EM, Geng X. Impact of repetitive transcranial magnetic stimulation on the directed connectivity of autism EEG signals: a pilot study. Med Biol Eng Comput. 2022;60(12):3655-64. https://doi.org/10.1007/s11517-022-02693-y.

[15] Desarkar P, Rajji TK, Ameis SH, Blumberger DM, Lai MC, Lunsky Y, et al. Assessing and stabilizing atypical plasticity in autism spectrum disorder using rTMS: Results from a proof-of-principle study. Clin Neurophysiol. 2022;141:109-18. https://doi.org/10.1016/j.clinph.2021.03.046.

[16] Zhang L, Li Q, Du Y, Gao Y, Bai T, Ji GJ, et al. Effect of high-definition transcranial direct current stimulation on improving depression and modulating functional activity in emotion-related cortical-subcortical regions in bipolar depression. Journal of affective disorders. 2023;323:570-80. https://doi.org/10.1016/j.jad.2022.12.007.

[17] Hoven M, Schluter RS, Schellekens AF, van Holst RJ, Goudriaan AE. Effects of 10 add-on HF-rTMS treatment sessions on alcohol use and craving among detoxified inpatients with alcohol use disorder: a randomized sham-controlled clinical trial. Addiction. 2023;118(1):71-85. https://doi.org/10.1111/add.16025.

[18] da Silva TR, de Carvalho Nunes HR, Martins LG, da Costa RDM, de Souza JT, Winckler FC, et al. Non-invasive Brain Stimulation Can Reduce Unilateral Spatial Neglect after Stroke: ELETRON Trial. Ann Neurol. 2022;92(3):400-10. https://doi.org/10.1002/ana.26430.

[19] Lisanby SH, Husain MM, Rosenquist PB, Maixner D, Gutierrez R, Krystal A, et al. Daily left prefrontal repetitive transcranial magnetic stimulation in the acute treatment of major depression: clinical predictors of outcome in a multisite, randomized controlled clinical trial. Neuropsychopharmacology. 2009;34(2):522-34. https://doi.org/10.1038/npp.2008.118.

[20] Wu LL, Potenza MN, Zhou N, Kober H, Shi XH, Yip SW, et al. Efficacy of single-session transcranial direct current stimulation on

addiction-related inhibitory control and craving: a randomized trial in males with Internet gaming disorder. J Psychiatry Neurosci. 2021;46(1):E111-e8. https://doi.org/10.1503/jpn.190137.

[21] Wu LL, Potenza MN, Zhou N, Kober H, Shi XH, Yip SW, et al. A role for the right dorsolateral prefrontal cortex in enhancing regulation of both craving and negative emotions in internet gaming disorder: A randomized trial. Eur Neuropsychopharmacol. 2020;36:29-37. https://doi.org/10.1016/j.euroneuro.2020.04.003.

[22] Yang CC, Khalifa N, Völlm B. Excitatory repetitive transcranial magnetic stimulation applied to the right inferior frontal gyrus has no effect on motor or cognitive impulsivity in healthy adults. Behav Brain Res. 2018;347:1-7. https://doi.org/10.1016/j.bbr.2018.02.047.

[23] Kwon HJ, Lim WS, Lim MH, Lee SJ, Hyun JK, Chae JH, et al. 1-Hz low frequency repetitive transcranial magnetic stimulation in children with Tourette's syndrome. Neurosci Lett. 2011;492(1):1-4. https://doi.org/10.1016/j.neulet.2011.01.007.

[24] Bloch Y, Harel EV, Aviram S, Govezensky J, Ratzoni G, Levkovitz Y. Positive effects of repetitive transcranial magnetic stimulation on attention in ADHD Subjects: a randomized controlled pilot study. World J Biol Psychiatry. 2010;11(5):755-8. https://doi.org/10.3109/15622975.2010.484466.

[25] Weaver L, Rostain AL, Mace W, Akhtar U, Moss E, O'Reardon JP. Transcranial magnetic stimulation (TMS) in the treatment of attention-deficit/hyperactivity disorder in adolescents and young adults: a pilot study. J ect. 2012;28(2):98-103. https://doi.org/10.1097/YCT.0b013e31824532c8.

[26] Gómez L, Vidal B, Morales L, Báez M, Maragoto C, Galvizu R, et al. Low frequency repetitive transcranial magnetic stimulation in children with attention deficit/hyperactivity disorder. Preliminary results. Brain Stimul. 2014;7(5):760-2. https://doi.org/10.1016/j.brs.2014.06.001.

[27] Niederhofer H. Effectiveness of the repetitive Transcranical Magnetic Stimulation (rTMS) of 1 Hz for Attention-Deficit Hyperactivity Disorder (ADHD). Psychiatr Danub. 2008;20(1):91-2.

[28] Masuda F, Nakajima S, Miyazaki T, Tarumi R, Ogyu K, Wada M, et al. Clinical effectiveness of repetitive transcranial magnetic stimulation treatment in children and adolescents with neurodevelopmental disorders: A systematic review. Autism. 2019;23(7):1614-29. https://doi.org/10.1177/1362361318822502.

[29] Laskov O, Klírová M. Effects of deep transcranial magnetic stimulation (dTMS) on cognition. Neurosci Lett. 2021;755:135906. https://doi.org/10.1016/j.neulet.2021.135906.

eTable 3. Grading of Recommendations Assessments, Development and Evaluation (GRADE) assessment of the strength of evidence for standard weighted meta-analysis

Outcome	Design	Risk of bias	Indirectness	Inconsistency	Imprecision	Publication bias	Grade Quality
Sustained attention	RCTx5	No serious	No serious indirectness	No serious inconsistency	Serious imprecision	Serious publication bias	⊕⊕OO[4,5]
Memory	RCT x 3	No serious	No serious indirectness	Serious inconsistency	Serious imprecision	Serious publication bias	⊕OOO[4,5]
Processing speed	RCT x 3	No serious	No serious indirectness	No serious inconsistency	Serious imprecision	Serious publication bias	⊕⊕OO[4,5]
Executive function	RCT x 2	No serious	No serious indirectness	Serious inconsistency	Serious imprecision	Serious publication bias	⊕OOO[3,4,5]

*Risk of bias was estimated using Cochrane risk of bias , studies were classified as having low risk of bias if none of the domains above was rated as high risk of bias and three or less were rated as unclear risk; moderate if one was rated as high risk of bias or none was rated as high risk of bias but four or

more were rated as unclear risk, and all other cases were assumed to pertain to high risk of bias.

Down-graded due to: [1] risk of bias, [2] indirectness, [3.] Inconsistency, [4.] Imprecision, [5.] publication bias

GRADE Working Group grades of evidence:

-**High certainty**: We are very confident that the true effect lies close to that of the estimate of the effect

-**Moderate certainty**: We are moderately confident in the effect estimate: The true effect is likely to be close to the estimate of the effect, but there is a possibility that it is substantially different.

-**Low certainty**: Our confidence in the effect estimate is limited: The true effect may be substantially different from the estimate of the effect.
-**Very low certainty**: We have very little confidence in the effect estimate: The true effect is likely to be substantially different from the estimate of effect.

eFigure 1. Funnel plot – sustained attention

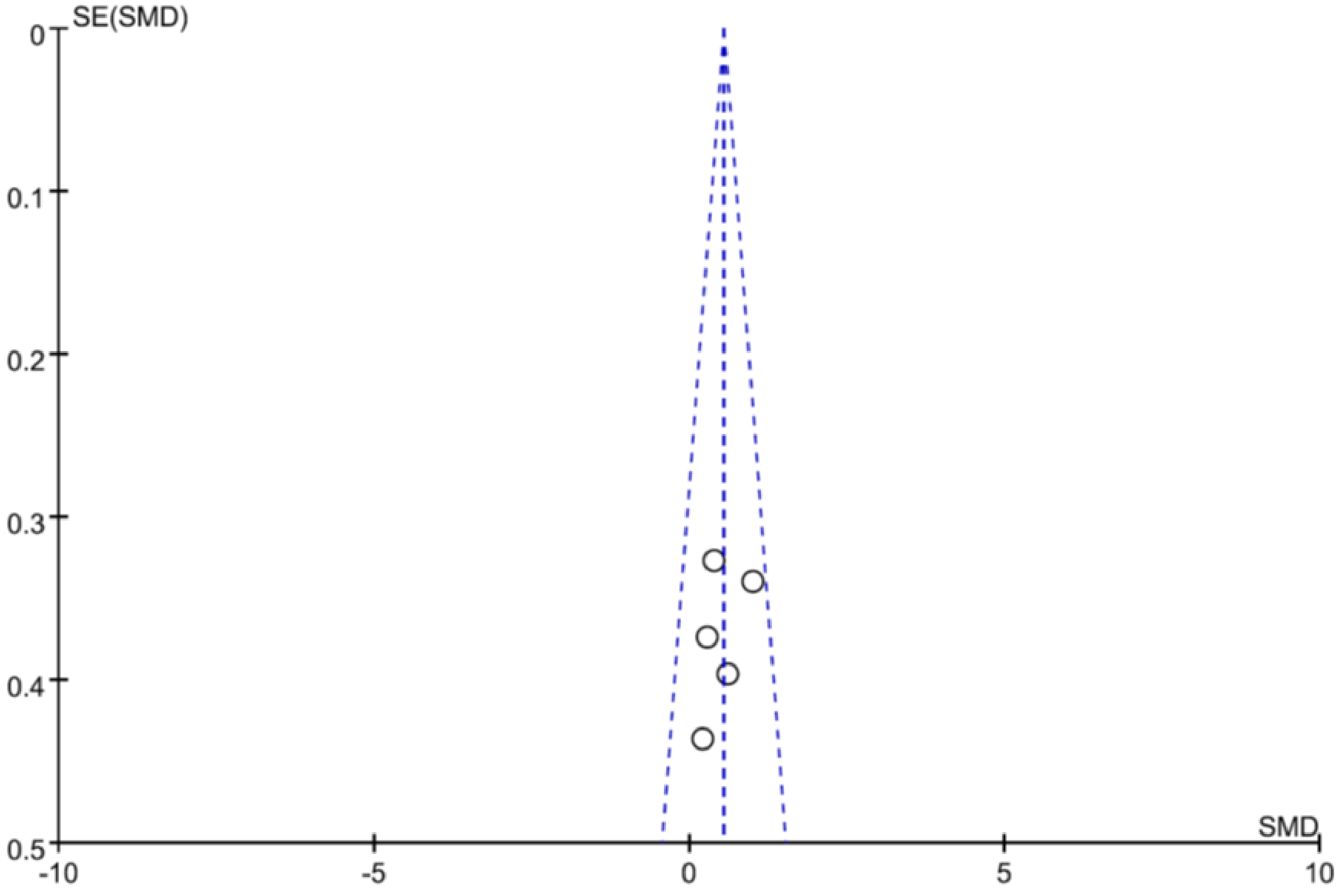

eFigure 2. Forest plot of effect size for comparing the difference in the improvement of processing speed between rTMS and control groups

Study or Subgroup	rTMS Mean	rTMS SD	rTMS Total	Control Mean	Control SD	Control Total	Weight	Std. Mean Difference IV, Random, 95% CI	Year
Cao P (2018)	4.16	1.1181	21	3.44	1.1181	19	33.9%	0.63 [-0.01, 1.27]	2018
Alyagon U (2020)	11.74	10.9993	15	5.56	10.91	14	24.9%	0.55 [-0.20, 1.29]	2020
Bleich-Cohen M (2021)	8.0935	8.5726	46	2.38	12.11	16	41.2%	0.59 [0.01, 1.17]	2021
**Total (95% CI)**			**82**			**49**	**100.0%**	**0.59 [0.22, 0.96]**	

Heterogeneity: Tau² = 0.00; Chi² = 0.03, df = 2 (P = 0.99); I² = 0%
Test for overall effect: Z = 3.13 (P = 0.002)

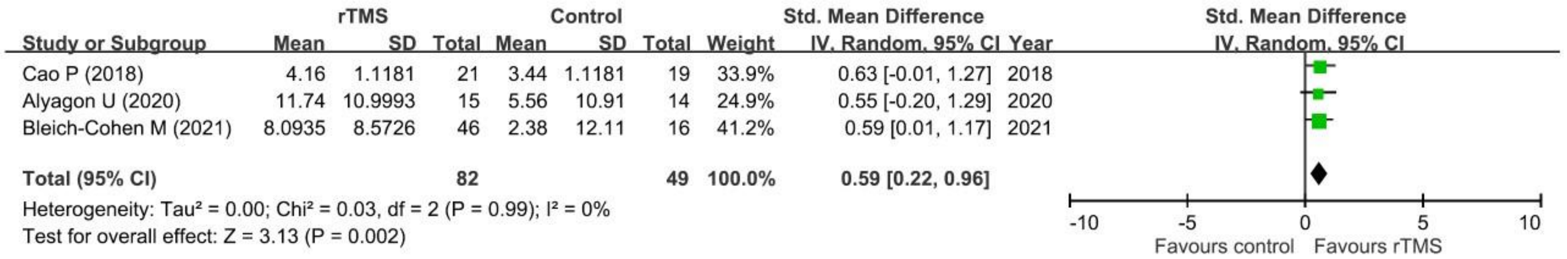

eFigure 3. Forest plot of effect size for comparing the difference in the improvement of memory between rTMS and control groups

	rTMS			Control group				Std. Mean Difference	
Study or Subgroup	Mean	SD	Total	Mean	SD	Total	Weight	IV, Random, 95% CI	Year
Cao P (2018)	1.15	0.9162	21	0.56	0.9162	19	33.7%	0.63 [-0.01, 1.27]	2018
Alyagon U (2020)	6.05	11.73	15	1.016	11.98	14	30.8%	0.41 [-0.32, 1.15]	2020
Bleich-Cohen M (2021)	4.617	8.946	46	9.2	12.13	16	35.5%	-0.46 [-1.03, 0.12]	2021
**Total (95% CI)**			**82**			**49**	**100.0%**	**0.18 [-0.52, 0.87]**	

Heterogeneity: $Tau^2 = 0.27$; $Chi^2 = 6.98$, df = 2 (P = 0.03); $I^2 = 71\%$

Test for overall effect: Z = 0.49 (P = 0.62)

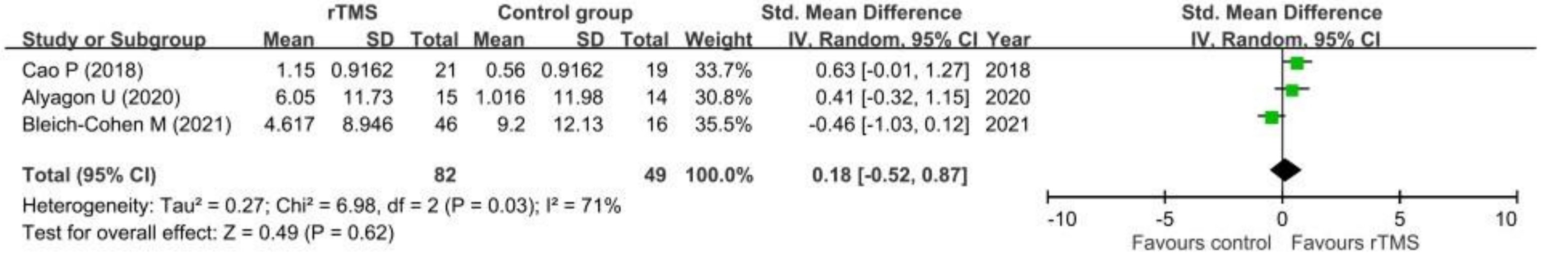

eFigure 4. Forest plot of effect size for comparing the difference in the improvement of executive function between rTMS and control groups

	rTMS			Control				Std. Mean Difference	
Study or Subgroup	Mean	SD	Total	Mean	SD	Total	Weight	IV, Random, 95% CI	Year
Cao P (2018)	21.57	5.7303	21	17.88	5.7303	19	33.6%	0.63 [-0.01, 1.27]	2018
Alyagon U (2020)	9.65	9.53	15	6.67	10.81	14	28.2%	0.28 [-0.45, 1.02]	2020
Bleich-Cohen M (2021)	1.7843	6.6351	46	2.87	7.41	16	38.2%	-0.16 [-0.73, 0.41]	2021
**Total (95% CI)**			**82**			**49**	**100.0%**	**0.23 [-0.24, 0.71]**	

Heterogeneity: Tau² = 0.07; Chi² = 3.31, df = 2 (P = 0.19); I² = 40%
Test for overall effect: Z = 0.96 (P = 0.34)

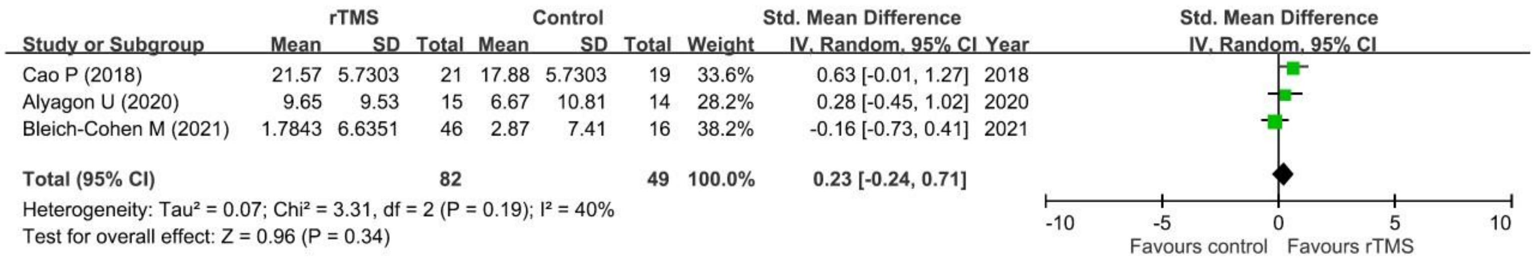

eFigure 5. Funnel plot – processing speed

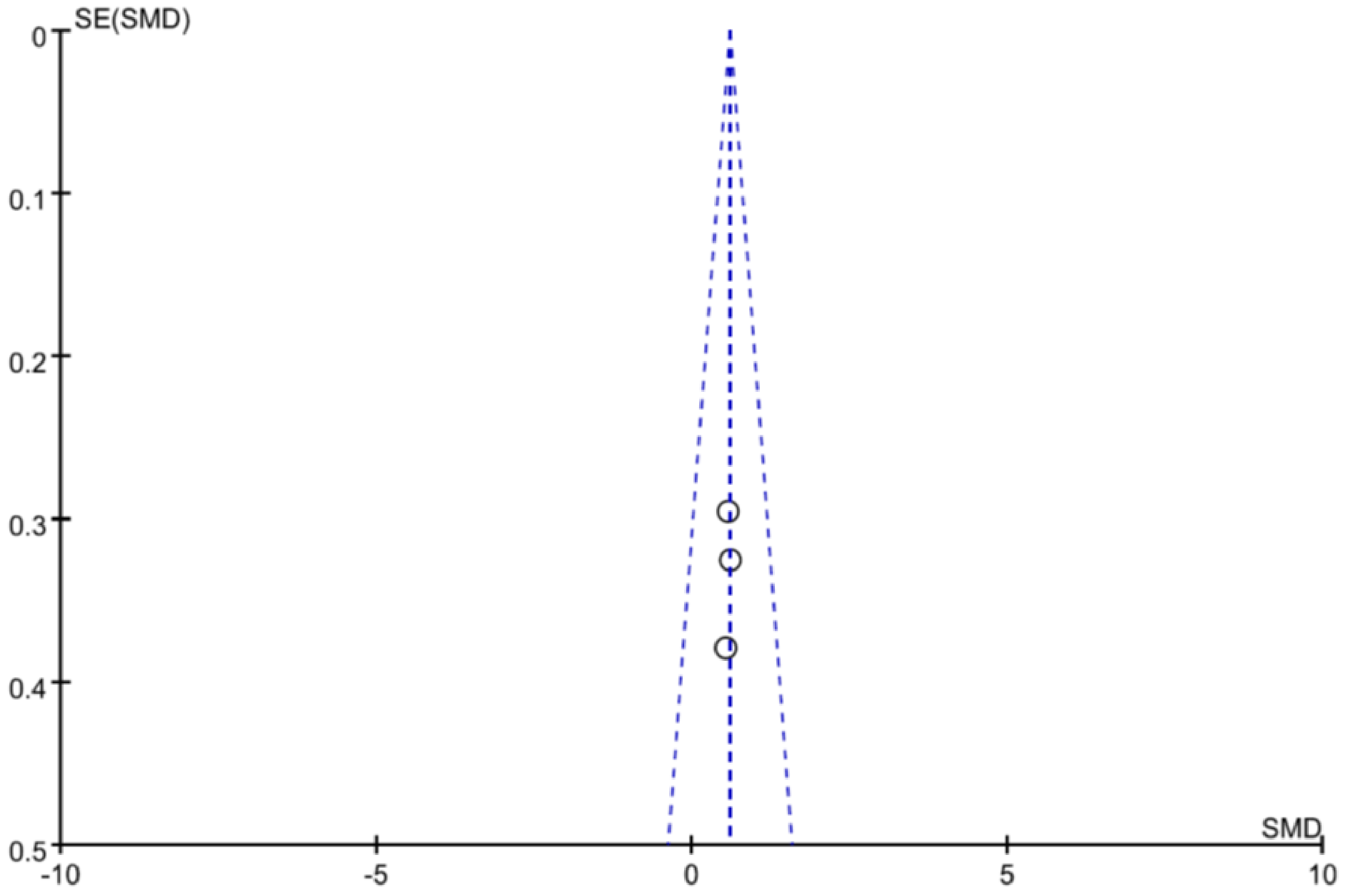

eFigure 6. Funnel plot – memory

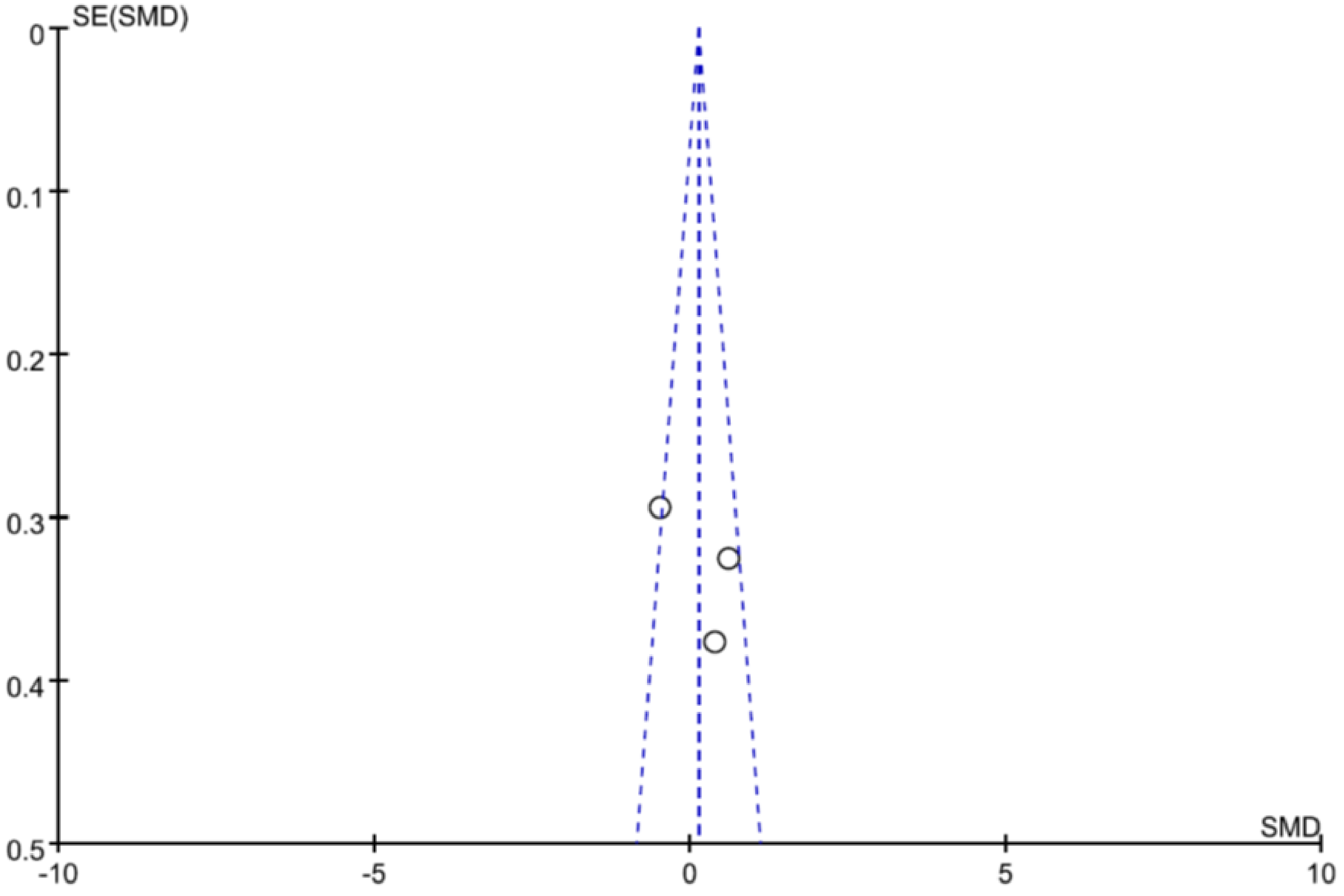

eFigure 7. Funnel plot – executive function

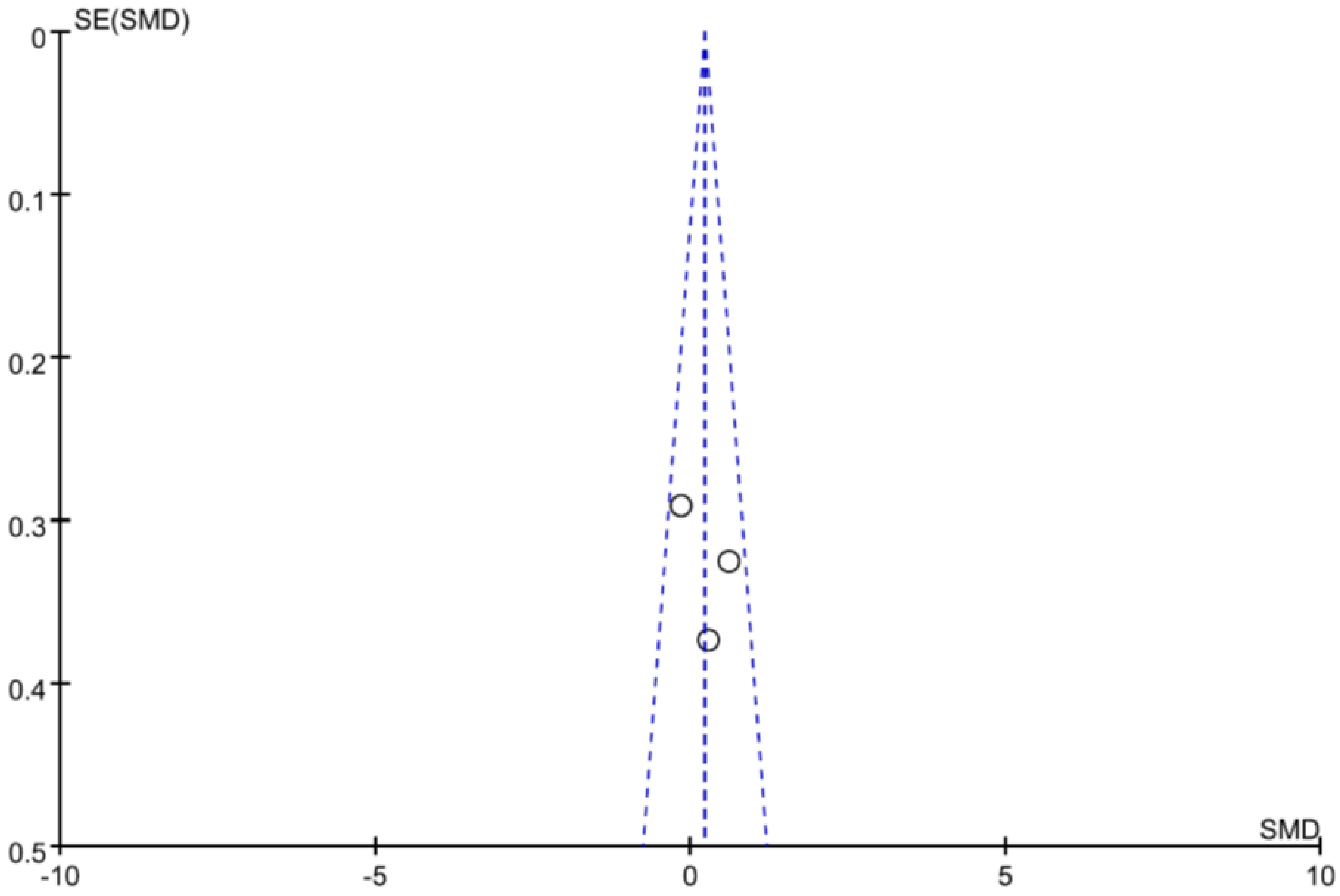

Supplement: Supplementary file 1 — Additional file 1. [file 12888_2023_5261_MOESM1_ESM.pdf]
